# Differential roles of the *Drosophila* EMT-inducing transcription factors Snail and Serpent in driving primary tumour growth

**DOI:** 10.1371/journal.pgen.1007167

**Published:** 2018-02-08

**Authors:** Kyra Campbell, Gaëlle Lebreton, Xavier Franch-Marro, Jordi Casanova

**Affiliations:** 1 Institut de Biologia Molecular de Barcelona (CSIC), Barcelona, Catalonia, Spain; 2 Institut de Recerca Biomèdica de Barcelona, (IRB Barcelona), The Barcelona Institute of Science and Technology (BIST), Barcelona, Catalonia, Spain; 3 Institute of Evolutionary Biology (CSIC-Universitat Pompeu Fabra), Functional Genomics and Evolution, Department Passeig Marítim de la Barceloneta, Barcelona, Spain; Harvard Medical School, Howard Hughes Medical Institute, UNITED STATES

## Abstract

Several transcription factors have been identified that activate an epithelial-to-mesenchymal transition (EMT), which endows cells with the capacity to break through basement membranes and migrate away from their site of origin. A key program in development, in recent years it has been shown to be a crucial driver of tumour invasion and metastasis. However, several of these EMT-inducing transcription factors are often expressed long before the initiation of the invasion-metastasis cascade as well as in non-invasive tumours. Increasing evidence suggests that they may promote primary tumour growth, but their precise role in this process remains to be elucidated. To investigate this issue we have focused our studies on two *Drosophila* transcription factors, the classic EMT inducer Snail and the *Drosophila* orthologue of hGATAs4/6, Serpent, which drives an alternative mechanism of EMT; both Snail and GATA are specifically expressed in a number of human cancers, particularly at the invasive front and in metastasis. Thus, we recreated conditions of Snail and of Serpent high expression in the fly imaginal wing disc and analysed their effect. While either Snail or Serpent induced a profound loss of epithelial polarity and tissue organisation, Serpent but not Snail also induced an increase in the size of wing discs. Furthermore, the Serpent-induced tumour-like tissues were able to grow extensively when transplanted into the abdomen of adult hosts. We found the differences between Snail and Serpent to correlate with the genetic program they elicit; while activation of either results in an increase in the expression of Yorki target genes, Serpent additionally activates the Ras signalling pathway. These results provide insight into how transcription factors that induce EMT can also promote primary tumour growth, and how in some cases such as GATA factors a ‘multi hit’ effect may be achieved through the aberrant activation of just a single gene.

## Author summary

Many cancer cells acquire abnormal motility behaviour leading to metastasis, the main cause of cancer related deaths. In many cancers, transcription factors capable of inducing motile migratory cell behaviours, so-called EMT transcription factors, are found highly expressed. However, the expression of these genes is not restricted to metastatic invasive cancers; they are often found in benign tumours, or in tumours long before they show any sign of metastasis. This observation motivated us to ask if they may play a role in driving primary tumour growth. Our results show that the *Drosophila* EMT-inducers Snail and Serpent are both capable of driving overproliferation. However, Snail overproliferation is accompanied by a decrease in cell size as well as cell death, and consequently the tissue does not increase in size. Serpent also drives cell proliferation but this occurs together with an increase in cell size, but not cell death, thus having a profound effect on the overall size of the tissue. We show that both Snail and Serpent trigger activation of the Yorki pathway and in addition Serpent, but not Snail, also triggers activation of the Ras pathway. These results provide insight into how activation of some EMT-inducing genes can also promote primary tumour growth.

## Introduction

Epithelial cells display a remarkable plasticity and while this property is vital for morphogenesis during normal embryonic development, there is now strong evidence supporting its critical role in tumour progression (reviewed in [1–3]). Several core inducers of the epithelial-to-mesenchymal transition (EMT) have been identified, such as the Snail/Slug family, Twist, SIP1, Zeb factors and Goosecoid, which effect EMT through the transcriptional repression of E-Cadherin. Our lab identified an alternative mechanism, whereby GATA factors affect cell plasticity through the modulation of apicobasal polarity [4, 5]. Similar to other EMT transcription factors, gain of function mutations in GATA factors have been found in a number of human cancers including breast, ovarian, pancreatic and colorectal cancer (CRC), and in metastases, such as liver metastasis from CRC patients [6–9].

Activating an EMT program promotes tumour invasion and metastasis, as it endows cells with the ability to break through basement membranes and migrate away from their site of origin. However, as well as promoting tumour dissemination, there is also evidence implicating EMT transcription factors in promoting primary tumour growth (reviewed in [10]). For example EMT transcription factors are often expressed in non-invasive tumours suggesting that they might promote oncogenic functions within the primary lesion, affecting tumour development long before the initiation of the invasion-metastasis cascade. Furthermore, Snail (Sna) interference in carcinoma cells dramatically decreases tumour growth, with tumours adopting a smaller size and having a decreased proliferation [11]. However, the effects of EMT transcription factors on primary tumour growth remain poorly characterised and many questions remain. Is activation of an EMT transcription factor alone sufficient to drive primary tumour growth, or just a step in the progression of the tumour? Are the mechanisms of driving growth shared by all EMT transcription factors?

To investigate this we have focused our studies on a classic EMT inducer Sna, and on Serpent (Srp), the *Drosophila* orthologue of hGATAs4/6, and modelled the effects of creating gain-of-function conditions in the wing imaginal discs of the fly. Due to the work of many laboratories, wing discs are now a well-established system for modelling epithelial tumours (see [12]), where the effects of proto-oncogenes can be analysed in a proliferating epithelium surrounded by a mature basement membrane. Here we show that activation of either Sna or Srp induces an increase in the proliferation of wing disc cells, together with tissue disorganisation and a loss of epithelial polarity. However, Sna-induced proliferation is accompanied by both a decrease in average cell size and extensive cell death, and consequently no overall increase in tissue size is observed. Conversely, Srp drives an increase in cell size as well as cell proliferation, leading to a large overall increase in the size of the wing disc. Furthermore, these Srp-induced tumor-like tissues are able to grow extensively when transplanted into the abdomen of adult hosts. We carried out a genetic screen of candidate genes to gain insight into the mechanisms underlying Srp-induced tissue growth and found that both the Yorki (Yki) and Ras pathways contribute to the phenotype. Our results show that while both Sna and Srp activation results in an increase in the expression of Yki target genes, Srp additionally activates Ras pathway signaling. Thus activation of an EMT transcription factor alone is sufficient to drive primary tumour growth, but this effect is transcription factor dependent. These results highlight the diverse consequences of reactivation of EMT transcription factors, which are often key developmental genes, and how a ‘multi hit’ effect may be achieved through the aberrant activation of just a single gene.

## Results

### Sna and Srp both drive EMT when ectopically expressed in the wing imaginal disc

**Fig 1:**
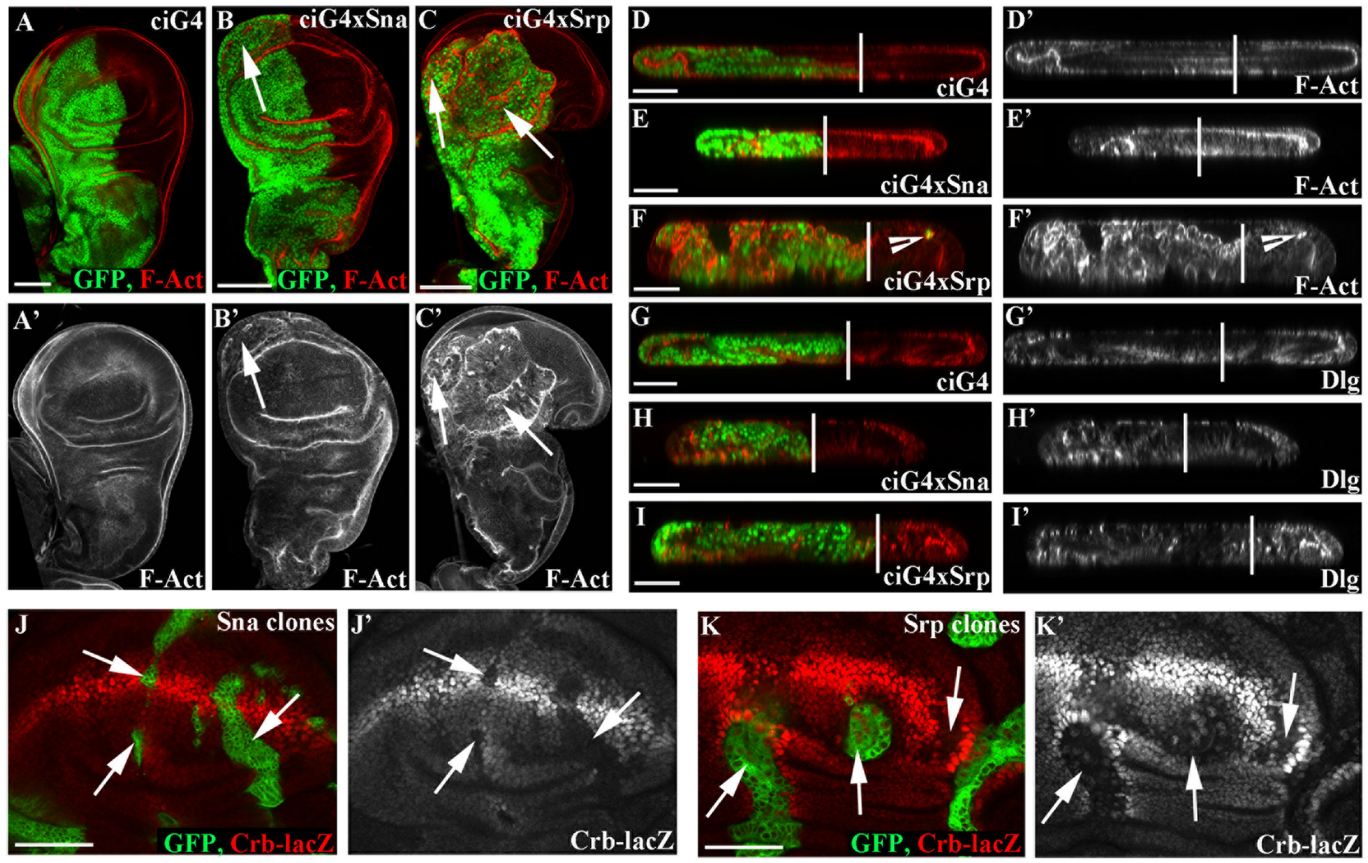
Both Srp and Sna drive an EMT in wing disc cells. A-I Staining for polarity markers in control *ci-Gal4*, *UAS-GFP; tub-Gal80*^*TS*^ discs (A, D, G); in *ci-Gal4*, *UAS-GFP*, *UAS-sna;tub-Gal80*^*TS*^ discs (B, E, H) and in *ci-Gal4*, *UAS-GFP*, *UAS-srp;tub-Gal80*^*TS*^ discs (C, F, I) 48 hours after shifting to 29°C, the permissive temperature. Staining for F-Act (A-F) and Dlg (G-I) in xy (A-C) and yz sections (D-I) shows that actin becomes delocalised in wing disc cells (arrows), and cells lose their regular columnar shape. Upon *srp*-overexpression some cells also appear in the posterior compartment (F, arrowhead). Vertical lines in D-I demarcate the anterior-posterior boundary J-K Clones of either *UAS-sna* (J) or *UAS-srp* (K) generated in a *crb*-LacZ reporter background. Clones were induced by a 30 min heat shock and fixed after 48 hours. Staining for LacZ showed that Crb is downregulated at the transcriptional level by both Sna and Srp (J, K, arrows). Scale bars A-C—100μm; D-K—50μm.

The *Drosophila* primordia of adult wing structures, the wing imaginal discs, are epithelial monolayers that actively proliferate during larval development to give rise to a 1,000-fold increase in the number of cells and tissue size. They are now widely used to study and dissect the basic mechanisms underlying different stages of epithelial tumour progression (for a review see [12]). We therefore decided to exploit this system to assess the consequences of generating gain-of-function conditions of the Sna and Srp transcription factors. To this end, we made use of the Gal4/UAS technique [13] and used *Ci-Gal4* to drive expression of either *sna* or *srp* in the anterior wing compartments and compared these with the wild type posterior compartments in the same discs (Fig 1A–1C); or *nub-Gal4*, which drives expression throughout the wing pouch (S1 Fig). Regional expression of either *sna* or *srp* causes tissue disorganization characteristic of an EMT, including loss of cell polarity, multilayering and loss of epithelial architecture (Fig 1A–1I, S1A–S1G Fig). F-actin becomes completely disorganised, particularly for Srp (Fig 1B, 1C, 1E and 1F, arrows, S1E and S1G Fig) and cell polarity proteins such as Dlg lose their tight localisation (Fig 1H and 1I, S1D–S1G Fig). In addition we see small numbers of Srp expressing cells in the posterior compartment (Fig 1F, arrowhead), pointing to Srp-expressing cells gaining the migratory and invasive properties associated with EMT.

The effects of Sna and Srp on tissue organisation vary notably, depending on the region of the wing disc, with the effects of Sna relatively restricted to the peripodial membrane (Fig 1B, arrow) and of Srp more pronounced in both the wing disc proper and peripodial membrane (Fig 1C, arrows). Interestingly, a recent study highlighted a susceptibility of certain areas of the discs to particular tumorigenic stimuli [14], thus these observations suggest either that ectopic activation of Sna and Srp produce the same effects but at different strengths, or that Sna and Srp are affecting different pathways.

Both Sna and Srp have previously been shown to impinge on apicobasal polarity in the *Drosophila* embryo through the direct transcriptional repression of the key cell polarity regulator Crumbs (Crb) [4, 15]. To see if this is also the case in the wing disc, we next made clones of Sna or Srp expressing cells in a background where wing disc cells have a *lacZ* enhancer trap insertion in the *crb* regulatory region (*crb-lacZ* [16]*)*. We find that *crb* transcription is repressed upon overexpression of either Sna or Srp (Fig 1J and 1K, arrows), indicating that, as in the embryo, Sna and Srp repress *crb* transcription. However, in contrast to the embryo, repression of *crb* alone in wing disc cells is not sufficient to perturb apico-basal polarity [17], and thus Sna and Srp likely affect the transcription of other genes regulating polarity in addition to *crb*, as seen during their embryonic roles in mesoderm [15] and midgut development [4].

### Srp drives neoplastic-like growth when expressed in the wing disc

**Fig 2:**
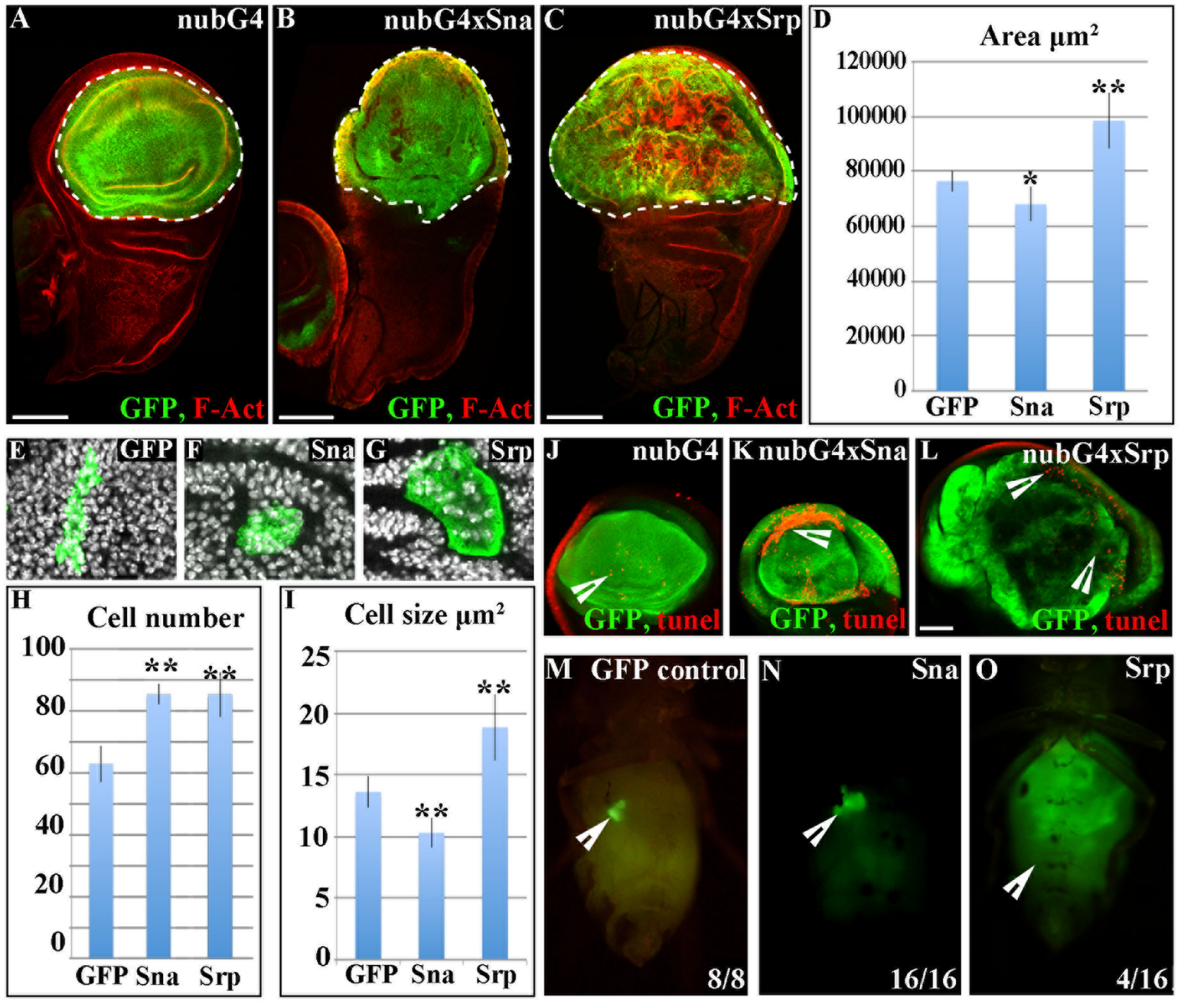
Srp drives an overgrowth of the wing disc. A-D, J-L Ectopic expression of either GFP alone (A, J), GFP and *sna* (B, K) or GFP and *srp* (C, L) in the wing disc; transgenes are under the control of *nub-Gal4* driver and *tub-Gal80*^*TS*^ and 3rd instar disc are fixed 48 hours after shifting to 29°C. Discs are stained for GFP (green) and F-act (red). Expression of *sna* results in a small, but significant decrease in wing disc size (B, D), whereas *srp* drives a large increase in the size of the GFP expressing compartment (C, D). E-G Clones of cells expressing either GFP alone (E), GFP and *sna* (F) or GFP and *srp* (G) were induced by a 30 min heatshock, and discs were fixed 48 hours after induction. H Counts of the number of cells in each clone showed that there is a significant increase in the number of cells in clones with either *srp* or *sna* expression. I Measuring the size of cells in clones of each condition revealed that in contrast to control clones, *srp*-expressing cells show an increase in both the magnitude and variability of their size. In contrast, *sna* expression shows a small but significant decrease in cell size. Data are presented as mean ± standard deviation (SD), *P<0.05, **P<0.005; paired t-test. J-L TUNEL staining reveals that Sna (K) but not Srp (L) causes a dramatic increase in cell death. M-O Adult fly micrographs taken 21 d after implantation of GFP-labeled larval wing tissue expressing GFP alone (M), GFP and *sna* (N) or GFP and *srp* (O). Ratios show the reproducibility of the phenotype are shown. Scale bars A-C—100μm; J-L—50μm.

Loss of *crb* has previously been shown to affect growth of *Drosophila* imaginal cells [17, 18], thus these results indicated that EMT transcription factors might impinge on growth in addition to tissue organisation. To investigate this further we drove expression of GFP together with Sna or Srp throughout the wing disc pouch and distal hinge for 48 hours using *Nub-Gal4*, and then measured the size of the GFP expressing area in 3^rd^ instar larvae. We found that overexpression of Srp induced a dramatic increase in the size of the tissue (Fig 2A–2D). Whereas, overexpression of Sna leads to a small, but significant decrease in tissue size (Fig 2A–2D).

Surprisingly, we found that Srp expression leads to heterogeneous effects, even within a single region of the wing disc, with a large number of cells appearing larger than in wild type (Fig 2C). As this suggested that tissue growth could be coming from an increase in cell size rather than overproliferation, we investigated this further by generating clones of cells expressing Sna or Srp and compared the number and size of cells within the clones with wild type clones generated concurrently (Fig 2E–2I). This analysis showed that Srp, and surprisingly also Sna, drives an increase in the average cell number within the clone (Fig 2H). In addition Srp drives a large increase in cell size in a very hetrogenous manner (Fig 2I). In contrast, Sna overproliferation is accompanied by a decrease in the average cell size (Fig 2I).

To further understand what is contributing to the phenotypes observed upon over-expression of Sna or Srp, we carried out TUNEL staining, to investigate a possible role for cell death. We found that overexpression of Sna causes massive cell death in certain regions of the wing disc proper (Fig 2K), whereas ectopic Srp does not cause a considerable increase in cell death (Fig 2L). Taken together, our experiments suggest that while Sna-induced cell proliferation occurs concurrently with cell death and a decrease in cell size, Srp induces both cell proliferation and cell growth in the absence of an increase of cell death. Thus the overall effect of ectopic Sna is a slight reduction in tissue size; in contrast overexpression of Srp leads to considerable tissue overgrowth.

To understand the contribution of cell death to the *sna* phenotype, we decided to overexpress *sna* together with the baculovirus p35 protein which blocks the action of a wide range of caspases and in inhibits cell death [19, 20]. To assess this we drove *sna* expression in the anterior wing compartment using *ci-Gal4*, and compared the size of this compartment with the wild type posterior compartment in the same disc. When *sna* alone is overexpressed, the A/P ratio is reduced from 1.6 to 1, and *sna* together with GFP, the A/P ratio is reduced to 1.2 (S2A and S2C Fig). When *sna* is overexpressed together with p35, which blocks cell death, we no longer see a decrease in tissue size, and the A/P ratio is 1.6, similar to wild type (S2B and S2C Fig). Our clonal analysis shows that Sna drives an increase in cell proliferation which is coupled to a decrease in average cell size. Taken together, our results suggest that when cell death is blocked, Sna-induced overproliferation is accompanied by a decrease in cell size which compensate for each other in terms of overall tissue size, and thus no overall change is observed. Furthermore, these data suggest that it is not differences in cell division that contribute to the differences in tissue size observed upon overexpression of Srp or Sna. Rather it is their contrasting effects on cell death and on cell size that lead to these pronounced differences.

To further understand the effect of Srp overexpression, we tried to follow the growth of *srp* expressing tissues for a longer period of time, however larvae bearing imaginal discs with ectopic *srp* expression died after two days, hence it was not possible to follow their growth for a longer period. Thus, we further characterized the growth potential of the tissue by allograft cultures [21, 22]. Wing tissue expressing GFP alone or GFP together with either *sna* or *srp* was transplanted into the abdomen of adult females and maintained for a period of 21 days. Tissue expressing GFP alone did not grow (Fig 2M), as shown previously [23, 24], nor did they grow when expressing GFP together with *sna* (Fig 2N). Conversely, when expressed together with *srp* the tissue grew many times larger than the original piece of transplanted tissue (Fig 2O) and could also grow when transplanted again into a new host. Altogether, these results indicate that *srp* expression can induce neoplastic tissue growth by triggering cells to proliferate and grow indefinitely.

### Srp-induced overgrowth is partially rescued by reducing Ras and Yki pathway activity

**Fig 3:**
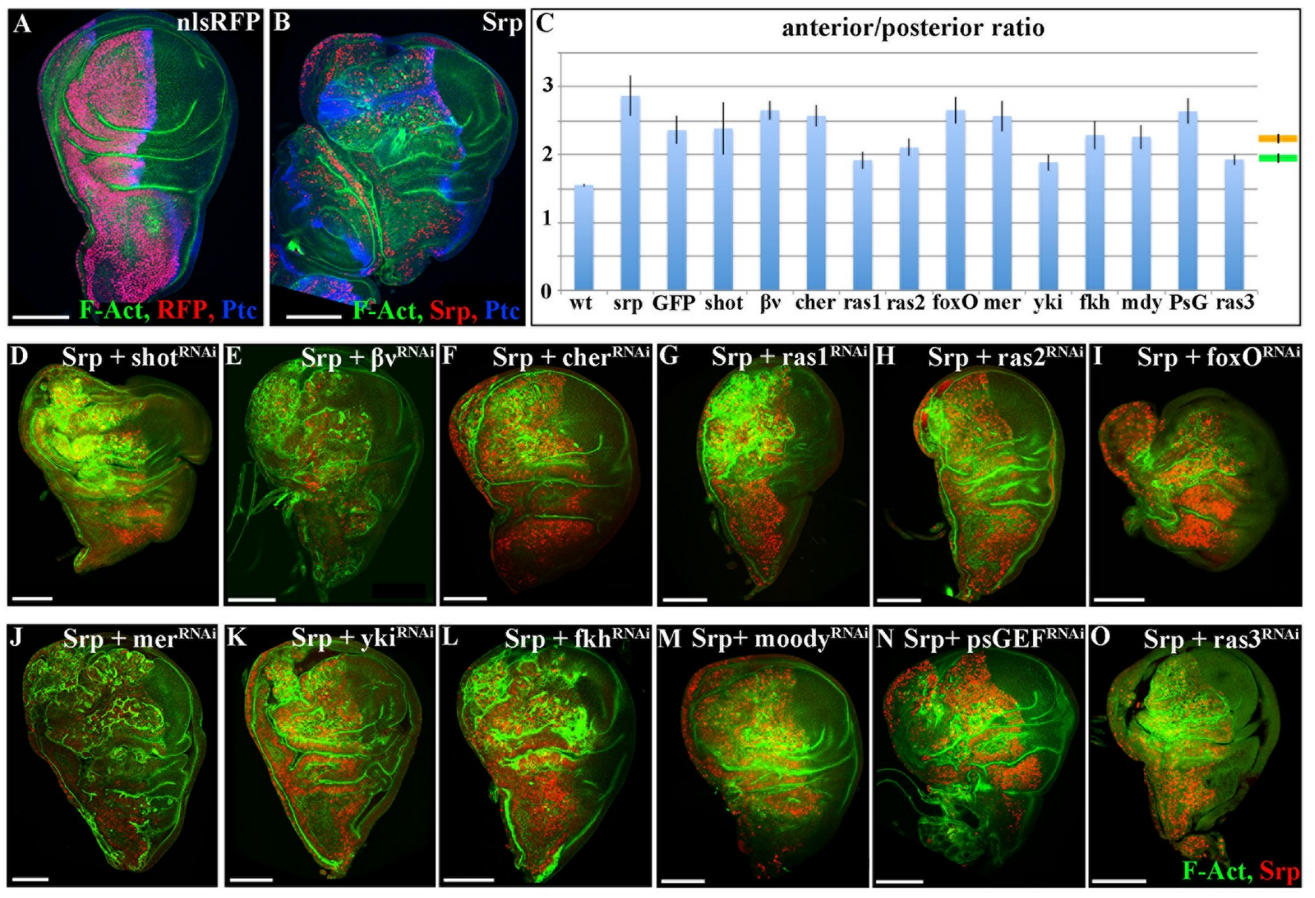
*srp*-induced overgrowth is partially rescued by RNAis against *ras* and *yki*. A, B, D-O 3rd instar wing discs from either control individuals expressing stingerRFP to mark anterior the anterior compartment (A) or individuals expressing the indicated transgenes in the anterior compartment (B, D-O). Discs are stained for F-Act (green), RFP (red) and Ptc (blue) (A); for F-Act (green), Srp (red) and Ptc (blue); and for F-Act (green) and Srp (red) (D-O). Ras1, ras2 and ras3 denote 3 independent RNAi transgenes against *ras*—see methods. Transgenes are under the control of the *ci-Gal4* driver and *tub-Gal80*^*TS*^ and the discs are fixed 48 hours after shifting to 29°C. Scale bars—100μm. C Histogram plotting the A/P width ratio of wing primordia expressing either nuclear RFP alone (wt), *srp* alone (Srp), *srp* together with GFP (GFP) or *srp* and dsRNA for the indicated genes under the control of *ci-Gal4* driver. Data are presented as mean ± SD. The mean ratio of discs expressing *srp* and an additional transgene is 2.49 with a standard error of the mean (SEM) of 0.05 and is depicted by the orange line; the mean ratio of discs showing a partial rescue is 1.96 with a SEM of 0.04 and is depicted by the green line. Scale bars—100μm.

**Fig 4:**
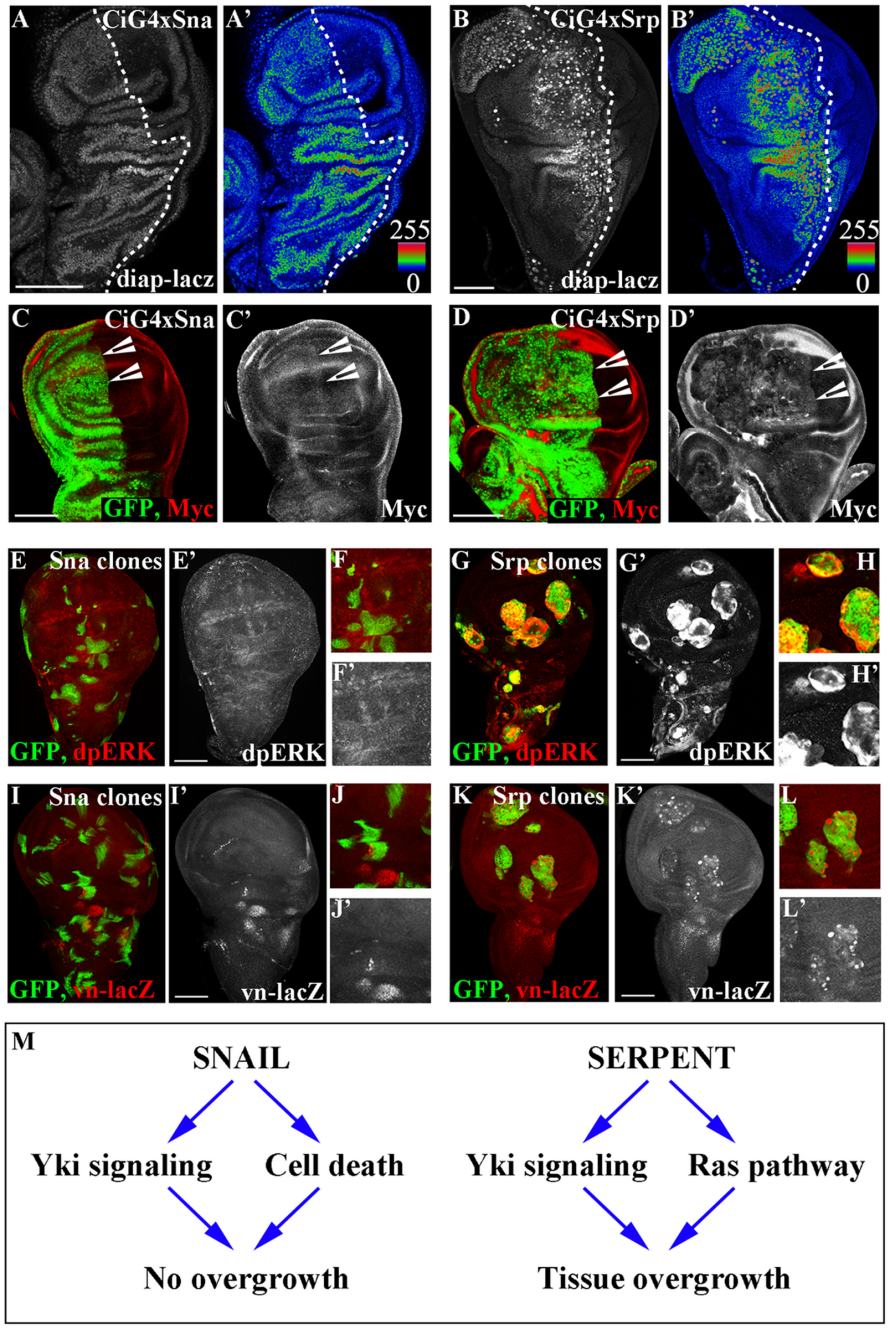
Srp activates both the Yki and Ras signalling pathways. A-D CiGal4, Gal80^ts^ was used to drive either *sna* (A, C) or *srp* (B, D) expression in the anterior compartment of discs containing the *diap*-lacZ reporter (A, B) or in a wildtype background together with GFP (C, D). Discs are stained for lacZ (A, B) or for GFP (green) and Myc (red) (C, D). E-K Clones of either UAS-*sna* (E, F, I, J) or UAS-*srp* (G, H, K, L) generated in either a wild type (E-H) or a *vn*-LacZ reporter background (I-L). Clones were induced by a 30 min heat shock and fixed after 48 hours. Staining for dpERK shows that Ras signalling is activated downstream of Srp (G, H), but not downstream of Sna (E, F). Staining for LacZ showed that *vn* is upregulated at the transcriptional level by Srp (K, L), but not by Sna (I, J). Scale bars A-K—100μm. (M) Model showing the effects of ectopic activity of Sna and Srp in the wing disc reported in this work (additional targets are likely to be triggered as well); Sna represses *crumbs*, which in turn activates Yki signalling; Srp represses *crumbs*, activating Yki signalling and also activates *vein*, which activates the RAS pathway via the EGFR.

To uncover the mechanisms underlying Srp-induced overgrowth, we decided to set up conditions for carrying out a genetic modifier candidate screen for genes that rescue this aspect of the Srp-phenotype. We drove expression of either a wild type nuclear label alone (Fig 3A), or of *srp* (Fig 3B) in the anterior wing compartment using *ci-Gal4*, and compared the size of this compartment with the wild type posterior compartment in the same disc (Fig 3A and 3B). We quantified the sizes of the two compartments as a measure of the ratio between the width of the anterior and posterior compartments in the same discs; while this ratio is 1.6 in wild type discs, the ratio rises to 3 upon *srp* expression in the anterior compartment (Fig 3C). We used this system to carry out a series of epistatic experiments with RNAis for candidate genes selected either by their role in signalling pathways involved in cell proliferation, or with a role related to EMT. For a big majority of the UAS-RNAis we found a general decrease of the Srp-induced oversize, compatible with the presence of another UAS construct being activated by the same GAL4 driver (Fig 3C, orange line; Fig 3D, 3E, 3F, 3I, 3J, 3L, 3M and 3N); a similar result obtained with a control UAS-GFP construct supports this interpretation (Fig 3C). Interestingly, 4 UAS-RNAi constructs gave rise to a greater reduction of the Srp-induced overgrowth three targeting *ras* and one targeting *yki*, both coding for well-known inducers of cell growth/proliferation (reviewed in [25]) (Fig 3C, green line; Fig 3G, 3H, 3K and 3O). Indeed, we found that ectopic expression of either *sna* or *srp* in the wing disc activated expression of *yki* downstream targets, as revealed by staining for the *diap1*-lacZ reporter construct [26] (Fig 4A and 4B), and the cellular growth promoter Myc [27] (Fig 4C and 4D). Since both *sna* and *srp* repress transcription of *crb*, and *crb* loss can cause Hippo pathway repression and Yki-dependent overproliferation [17, 18], it is likely that repression of *crb* by Sna or Srp is activating, or at least contributing, to the increase in the transcription of Yki target genes.

Thus, while Yki activity might be necessary for Srp-induced overgrowth it is clearly not sufficient, as Yki is also activated by ectopic expression of *sna*, which does not induce tissue overgrowth. Hence, we focused on the Ras pathway, the other element identified by the RNAi screen. Ras is a transducer of a phosphorylation cascade that conveys the activity of Receptor Tyrosine Kinases (RTK) resulting in the di-phosphorylation and activation of the extracellular signal-regulated kinase (dpERK) [28]. Once active, Erk phosphorylates its substrates in the cytoplasm or in the nucleus [29]. Again, we found that *srp* expressing clones in the wing disc activate the Ras pathway as reported by dpERK staining (Fig 4G). Conversely, there was no dpERK staining in *sna* expressing clones in the wing disc (Fig 4E).

Ras signalling has previously been reported to protect cells from cell death, in particular in tumourigenic situations which arise from a loss of cell polarity [30]. We therefore wondered if blocking Ras signalling together with overexpression of Srp would lead to an increase in cell death comparable to what we see with overexpression of Sna alone. However, when we stained discs overexpressing Srp together with *ras*-RNAi in the anterior compartment for Death caspase-1 (Dcp1), we did not see any increase in the levels of cell death when compared to the wildtype posterior compartment (S3). This indicates that Ras signalling is not contributing to growth simply through preventing stress-induced cell death, and likely cooperates with Yki to induce uncontrolled tissue growth as recently reported in [31].

We next investigated how Srp, but not Sna, could elicit the activation of the Ras pathway. Srp is a key regulator of embryonic midgut morphogenesis [4, 32], thus it was intriguing to note that EGFR signaling through activation of its ligand vein (vn) is required during embryonic midgut development [33]. *vn* codes for one of the ligands of the EGFR [34], an RTK widely expressed in *Drosophila*, including the wing disc [35, 36], and ectopic activation of Vn in the wing triggers Ras activation through the EGFR [37]. We tested for *vn* activation by generating clones of Sna or Srp expressing cells in a background where wing disc cells have a *lacZ* enhancer trap insertion in the *vn* regulatory region (*vn-lacZ*). We found a massive upregulation of *vn* transcription within *srp* expressing clones (Fig 4K), but not with *sna* expressing clones (Fig 4I). In addition, because Vn is a secreted ligand it can thus bind the EGFR, and hence activate the Ras pathway, in the cells close to the ones in which it is transcribed; thus, activation of *vn* transcription can also account for the non-autonomous activation of the Ras pathway by Srp as revealed by dpERK staining in cells close to the *srp* expressing clones (Fig 4H, S4 Fig, arrowheads). Taken together, these data indicate that activation of Srp leads to tissue overgrowth through the combined activation of Yki and Ras signalling pathways (Fig 4M). In contrast, while Sna also activates the transcription of Yki targets, it does not activate the Ras signalling pathway, which is likely to be one of the causes of the difference in the effects of Sna and Srp on the final tissue size (Fig 4M).

To assess the role of Ras activation in the Srp overexpression phenotype we planned to reduce *vn* function in the Srp overexpression background by using the available UAS-*vn*RNAi construct. Unfortunately, this experiment turned not to be simple because of the chromosome location of all the constructs required. Thus, as an alternative, we resorted to an UAS-*EGFR*RNAi construct to impair Ras activation. It is well known that EGFR signalling is normally required for wing development and in particular that impairing EGFR signalling in an otherwise wild type wing impairs the normal rate of proliferation and the wing size [36, 38]. Accordingly, impairment of EGFR signalling in a *srp* overexpression background not only reverts the overgrowth phenotype but generates wings smaller than wild type indicating an absolute requirement of EGFR-mediated Ras activation for *srp* overgrowth (S3B Fig).

## Discussion

EMT transcription factors are often found upregulated in human tumours (reviewed in [39]). Given their role in driving a transition from a polarised static epithelial cell to a migratory invasive cell state, much focus has been put on the pro-invasive and metastatic implications of their aberrant expression. However, tumour progression involves the progressive acquisition of many other biological capabilities including sustained proliferation, evasion of growth suppressors and resistance to cell death (reviewed in [40]), and there is increasing evidence suggesting that EMT transcription factors contribute to these earlier stages of tumour progression (reviewed in [10]). In this study we show that the *Drosophila* EMT-inducers Sna and Srp drive not only EMT, but also over-proliferation in a well-established epithelial tumour model. Sna-driven proliferation is accompanied by extensive cell death and a decrease in cell size, and thus the overall effects of aberrant Sna expression on tissue size are negligible. In contrast, Srp drives an increase in cell size as well as cell proliferation, but not cell death, leading to a profound overall increase in the size of the tissue, which is particularly evident upon transplantation, when the tissue has more time to grow. We find that both Sna and Srp repress *crb* transcription, which has previously been shown to induce a repression of the Hippo pathway and thus drive Yki-dependent overproliferation [17, 18]. Indeed, we show that both Sna and Srp activate Yki activity, which has previously been shown to drive excess proliferation in the wing disc. However, in addition to this, we find that Srp also activates the mitogenic Ras pathway, which has recently been shown to act synergictically with Yki to promote hyperproliferation and tumour development in the *Drosophila* wing disc [31]. Studies in breast cancer models and oesophageal epithelial cells have shown that the EMT transcription factors Twist and Zeb contribute to primary tumour growth through the activation of programs that prevent cells from undergoing oncogene-induced senescence and apoptosis [41–43]. Taken together with our results, this suggests that EMT transcription factors can contribute to the multistep process of tumour progression through the activation of different onco-promoting cell biological processes, and that this is both transcription factor and tumour dependent.

EMT transcription factors drive a loss of epithelial cell polarity, which has been shown to activate cell death pathways in a number of contexts. For example, *scribble* (*scrib*) mutant clones are completely eliminated from wild type discs through programmed cell death pathways [44]. Intriguingly, while overexpression of both Srp and Sna drives a loss of cell polarity, an increase in cell death is only seen with Sna, whereas Srp appears to correlate with an increase in cell survival. This is despite the fact that we find an increase in the transcription of the key apoptosis inhibitor Diap1 in both scenarios. Diap1 functions as an E3-ubiquitin ligase that protects cells from unwanted death by blocking the activity of the caspase DRONC and the *Drosophila* apoptotic protease-activating factor-1 (Apaf-1) homolog, Dark, and the relative levels of Diap1, Dronc and Dark are important in determining the outcome ie. cell survival vs cell death [45]. As we see a lot of cell death in the wing disc upon overexpression of Sna, despite a clear increase in Diap1 expression, this suggests that the levels of Diap1 induced are not sufficient to block the level of cell death induced by Sna. While we see a comparable increase in the levels of Diap1 upon Srp overexpression, Srp also activates Ras, which has been reported to protect cells with mutations in cell polarity genes from death [30]. However, reducing Ras signalling does not lead to an increase in cell death when Srp is overexpressed. We have previously see that ectopic Srp also induces expression of Forkhead (Fkh) [4], which has been reported to act as a survival factor in a number of *Drosophila* systems, including the midgut [46, 47]. These results therefore suggest that cells expressing ectopic Srp evade death through the upregulation of multiple cell survival factors.

Intriguingly, so-called EMT transcription factors such as Sna, Srp, Twist and Zeb proteins often activate many developmental pathways and processes of which a loss of cell polarity and EMT is only a part. They are all expressed in multiple tissues during development and play pleotrophic roles, depending on the context and time window in which they are activated. We want to emphasise the cell context dependence of the activity of these genes, which suggests that other genes may collaborate to the Srp and Sna induced transformations. For example, in *Drosophila* Sna activates an EMT in mesoderm cells during early stages of embryonic development [48], but later on it plays distinct roles during central nervous system [49] and peripheral eye development [50]. Srp is required for EMT in the *Drosophila* midgut, but also for midgut cell specification [48], and additionally plays multiple roles during specification and maturation of the haemocytes [51, 52]. Hence, it is not surprising that activation of such transcription factors outside the normal controls imposed during development can impinge on multiple cell features and signalling programs in addition to EMT, and thus play key roles in the initiation and development of primary tumours, rather than being limited to the steps of cancer cell invasion and metastatic spread. Additionally, it is worth noting that the effect of Srp activity on tissue overgrowth in the wing disc is due, at least in part, to the ectopic triggering of effector genes normally elicited by Srp in the midgut, one of its regular domains of expression.

We previously investigated the effects of triggering ectopic *sna* and *srp* in the *Drosophila* embryo, by driving their expression in ectodermal epithelial cells in which they are never normally expressed [53]. While we found that *sna* had no effect on ectoderm cell behaviour [53], a more recent study showed that when *sna* was expressed at high levels using a maternal driver, it triggers adherens junction disassembly in ectodermal cells, and in rare cases, the movement of some cells to inside the embryo [54]. Similarly, ectopic *srp* drives a loss of apicobasal polarity and junction disassembly, although with *srp* there is a profound migration of cells into the embryo. Remarkably, in the embryo we do not see any proliferation in these circumstances. Conversely, in wing discs we see overproliferation, but very little cell migration. Intriguingly, a "Go or Grow" hypothesis has been proposed which postulates that cell division and cell migration are temporally exclusive events and that tumor cells defer migration to divide and *vice versa* [55–59]. Our results suggest that EMT transcription factors can drive migration or proliferation, but tend to favour one over the other at any given time. Given the fact that EMT transcription factors are increasingly associated with cancer stem cells, it will be important to unravel when and how EMT promotes one over the other.

The transformation of a healthy cell into a cancerous one requires multiple mutations and cooperation between different oncogenic/tumor suppressor mutations. Not only can EMT transcription factors accelerate tumour progression by the activation of multiple biological processes, this can also be exacerbated through cooperative effects of the different pathways. For example, in breast cancer models cooperation between Twist and an active form of RAS is sufficient to trigger transformation of mammary epithelial cells into malignant cells exhibiting all the characteristic features of claudin-low tumors [60]. Similarly our results suggest that ectopic Sna in combination with situations where cells become resistant to cell death may have catastrophic effects. Remarkably, over-expression of Srp alone activates both the EGFR/Ras and Yki signalling pathways. Of note, over-activation of the Ras pathway in situations of compromised cell polarity often leads to dramatic tissue overgrowth, for example when oncogenic Ras is combined with a *scribble* mutation [44]. Furthermore, it has previously been shown that loss of *Drosophila* cell polarity regulators such as Scribble promotes epithelial tissue overgrowth and cooperation with the Ras pathway through impaired Hippo pathway signaling [61]. Thus the profound effects seen upon Srp activation are likely due to cooperation between these two pathways. GATA factors are increasingly found deregulated in human tumours, both at the invasive front and in primary lesions and are receiving increasing attention as onco-promoting genes (reviewed in [62]). Our work suggests that GATA factors could be activating multiple tumour promoting pathways, that act cooperatively both in early stages of primary tumour growth and later in driving invasion and metastasis.

## Methods

### Fly strains and genetics

Details for all genotypes and transgenes can be found in flybase (http://flybase.org) or in references listed here. Conditional activation of either RNAi or gene expression was achieved using the Gal4/Gal80^ts^ system [63]. To misexpress in the anterior compartment *Ci-Gal4; tubulinGal80*^*ts*^ was used, and in the wing disc proper *nub-Gal4*, *UAS-srcGFP*; tubulinGal80^ts^. Crosses were kept at 18 °C until late in L2 when larvae were shifted to 29 °C for 48 hours and dissected. Gal4 lines were crossed to the following UAS transgenes: *UAS-srp* (from D. Hoshizaki), *UAS-sna* (from J. Kumar); *UAS-shot*^*RNAi*^ (#41858), *UAS-cheerio*^*RNAi*^ (#35755), UAS-EGFR^RNAi^ (#25781), *UAS-ras*^*RNAi*^ (ras1—#29319, ras2—#34619, ras3—#35414), *UAS-foxO*^*RNAi*^ (#27656), *UAS-merlin*^*RNAi*^ (#28007), *UAS-fkh*^*RNAi*^ (#33760), *UAS-moody*^*RNAi*^ (#36821), *UAS-psGEF*^*RNAi*^ (#33433) from Bloomington stock center; UAS-*βvintegrin*^*RNAi*^ (#2503), *UAS-yki*^*RNAi*^ (#40497) from VDRC. Clones were generated by crossing ywhsflp; *Tub>y+>gal4*, *UAS GFP* to *UAS-sna or UAS-srp*, and larva were heat-shocked for 30 mins and fixed at 3^rd^ instar after 48 hours. The following reporter lines were used: *crb*^*M11*.*M2*^ (crb-lacZ, from M.Alverof [16]), diap-lacZ [26] and vn^rF264^ (vn-lacZ).

### Immunohistochemistry, fixed image acquisition and analysis

The following antibodies used were: rabbit anti-aPKC (1:500; Santa Cruz); rat anti-crb (1:500; gift from E.Knust); mouse anti-Dlg (1:500; Hybridoma Bank); rabbit anti-dpERK (1:200; Cell signalling); goat anti-GFP (1:500; Abcam); rabbit anti-RFP (1:300; Life Technologies); Phalloidin TRITC (1:200; Sigma Aldrich); mouse anti-Patched (1:100; Hybridoma Bank); rat anti-Srp (1:500 made in the Casanova lab). The TUNEL assay was performed using the *In situ* Cell Death Detection Kit (Roche). Cy2, Cy3 and Cy5-conjugated secondary antibodies were from Molecular Probes and were used at 1:200 dilutions, and discs were mounted in Vectashield containing DAPI. Confocal images were acquired with a Leica SP5. Images were analysed with Fiji software [National Institutes of Health (NIH) Bethesda, MD] and assembled into figures using both Fiji and the Adobe Photoshop software.

### Allograft transplantations

Wing discs with *nub-Gal4*, *UAS-srcGFP*; tubulinGal80^ts^ alone, or crossed to UAS-*sna* or UAS-*srp* were dissected from third instar larvae that had been kept at 18 °C until late in L2 and shifted to 29 °C for 48 hours, and transferred to cold PBS. Discs were fragmented into small pieces using Vannas scissors (World precision Instruments) and implanted into the abdomens of virgin females, as described in [22]. Fly hosts were kept at 25 °C and examined 21 d post-implantation.

### Quantification of tissue growth

For measuring the effects on the wing disc proper, *nub-Gal4*, *UAS-srcGFP*; tubulinGal80^ts^ was crossed to either UAS-*sna* or UAS-*srp*. Crosses were kept at 18 °C until late in L2 when larvae were shifted to 29 °C for 48 hours and dissected. The size of the GFP expressing area was then measured using Fiji software. For Anterior Posterior size ratios, the maximal width of the anterior compartment was divided by that of the posterior, again measured using Fiji software.

## Supporting information

S1 Fig (Related to Fig 1). Overexpression of Sna or Srp induces a loss of cell polarity. A-G Staining for polarity markers in control (A), nub-Gal4, UAS-Sna; tub-Gal80^TS^ discs (B, D, E) and in nub-Gal4, UAS-Srp; tub-Gal80^TS^ discs (C, F, G) 48 hours after shifting to 29°C, the permissive temperature. Staining for Crb (A-C) shows that Crb is lost from the *nub* expressing region upon ectopic Sna or Srp expression (B, C—dotted red lines denotes *nub* expressing region). D, E Staining for F-Act and Dlg show that cells towards the edges of the Sna expressing regions lose polarity (D, E, arrowheads). F, G Srp overexpression drives a dramatic loss of cell polarity throughout the *nub* expressing region, as seen by staining for F-Act and Dlg (F, G, arrowheads) Scale bars—100μm.
(TIF)

S2 Fig (Related to Fig 2). The decrease in tissue size seen upon Sna overexpression is largely due to cell death. A, B, 3rd instar wing discs from either individuals expressing GFP to mark anterior the anterior compartment together with Sna (A) or together with both Sna and p35, which blocks cell death (B). Discs are stained for GFP (green) and F-Act (red). Transgenes are under the control of the ci-Gal4 driver and tub-Gal80^TS^ and the discs are fixed 48 hours after shifting to 29°C. Scale bars—100μm. (C) Histogram plotting the A/P width ratio of wing primordia expressing either GFP alone (wt), Sna alone (sna), Sna together with GFP (sna, GFP) or Sna together with both GFP and p35 (sna, GFP, p35). Data are presented as mean ± SD. *P<0.005; paired t-test. There is no significant difference between wt and sna, GFP, p35.
(TIF)

S3 Fig (Related to Fig 3). A-C 3rd instar wing discs from either individuals expressing GFP to mark anterior the anterior compartment together with Srp (A, C) and RasRNAi (A); or just Srp (B). Transgenes are under the control of the ci-Gal4 driver and tub-Gal80^TS^ and the discs are fixed 48 hours after shifting to 29°C. A Staining for Dcp1 to visualise cell death. D. Histogram plotting the A/P width ratio of wing primordia expressing either GFP alone (wt), Srp alone (srp) or Srp together with EGFR^RNAi^. Data are presented as mean ± SD. *P<0.005; paired t-test.
(TIF)

S4 Fig (Related to Fig 4). Srp drives expression of dpERK both inside and outside of clones. (A) Clones of UAS-*srp* were generated in an otherwise wild type background. Clones were induced by a 30 min heat shock and fixed after 24 hours. Staining for dpERK shows that Ras signalling is activated both inside (arrow) and outside (arrowhead) clones.
(TIF)
