# Supplementary figures and images for: Differential roles of the Drosophila EMT-inducing transcription factors Snail and Serpent in driving primary tumour growth
Source: PLoS Genet. 2018 Feb 8;14(2):e1007167. doi: 10.1371/journal.pgen.1007167 (PMC5821384; doi:10.1371/journal.pgen.1007167)

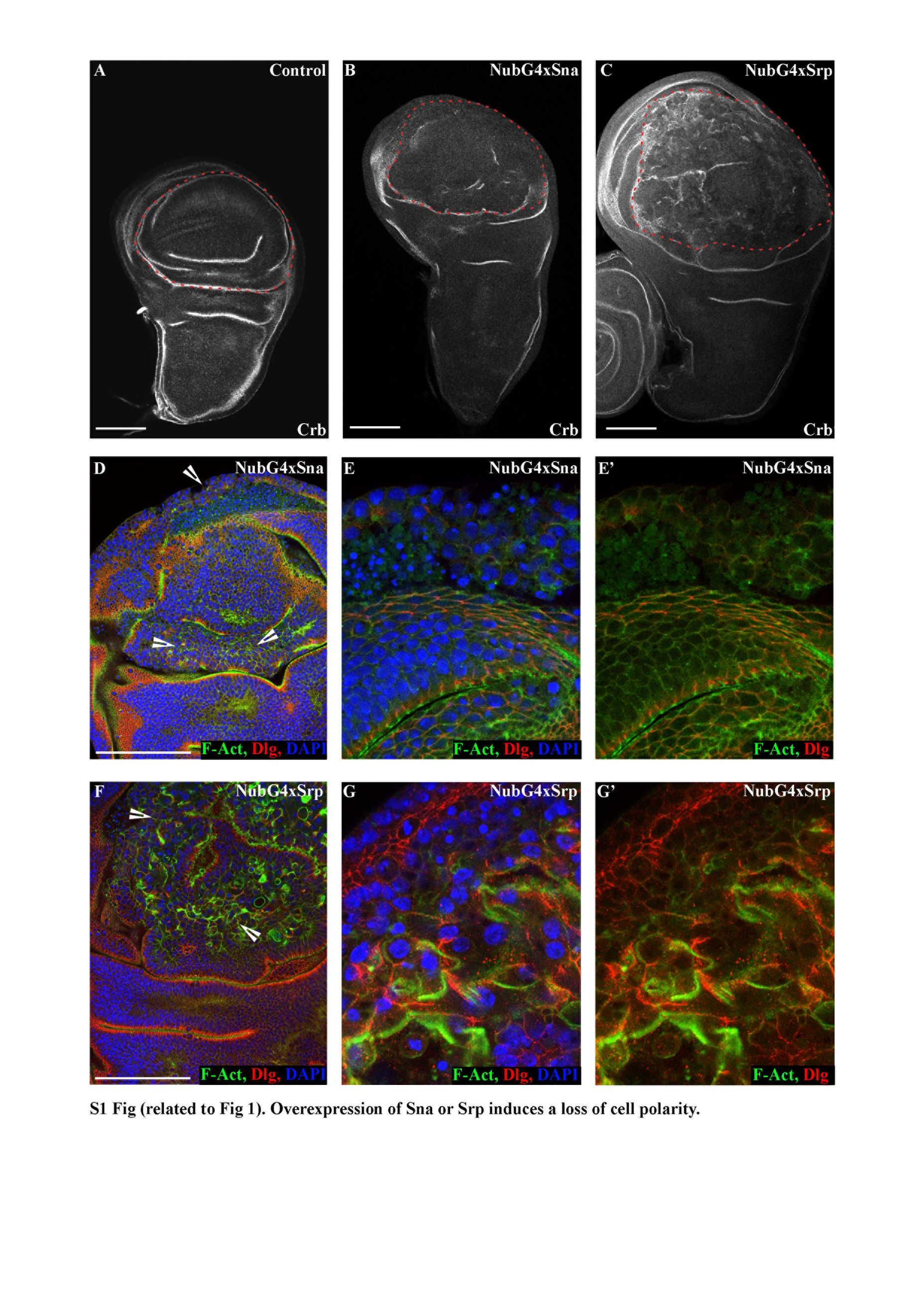

Supplement: S1 Fig — A-G Staining for polarity markers in control (A), nub-Gal4, UAS-Sna; tub-Gal80TS discs (B, D, E) and in nub-Gal4, UAS-Srp; tub-Gal80TS discs (C, F, G) 48 hours after shifting to 29°C, the permissive temperature. Staining for Crb (A-C) shows that Crb is lost from the nub expressing region upon ectopic Sna or Srp expression (B, C—dotted red lines denotes nub expressing region). D, E Staining for F-Act and Dlg show that cells towards the edges of the Sna expressing regions lose polarity (D, E, arrowheads). F, G Srp overexpression drives a dramatic loss of cell polarity throughout the nub expressing region, as seen by staining for F-Act and Dlg (F, G, arrowheads) Scale bars—100μm. (TIF) [file pgen.1007167.s001.tif]

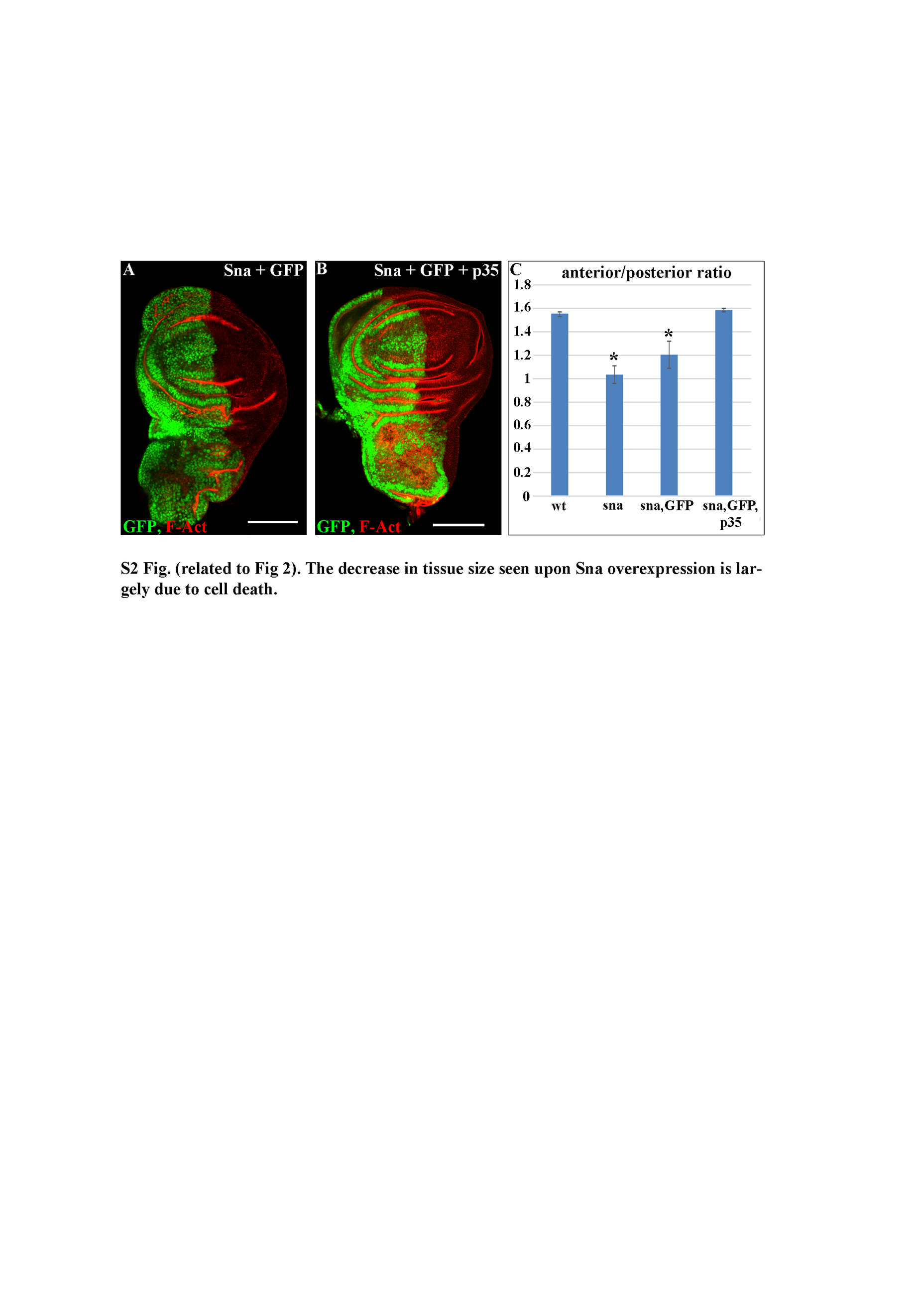

Supplement: S2 Fig — A, B, 3rd instar wing discs from either individuals expressing GFP to mark anterior the anterior compartment together with Sna (A) or together with both Sna and p35, which blocks cell death (B). Discs are stained for GFP (green) and F-Act (red). Transgenes are under the control of the ci-Gal4 driver and tub-Gal80TS and the discs are fixed 48 hours after shifting to 29°C. Scale bars—100μm. (C) Histogram plotting the A/P width ratio of wing primordia expressing either GFP alone (wt), Sna alone (sna), Sna together with GFP (sna, GFP) or Sna together with both GFP and p35 (sna, GFP, p35). Data are presented as mean ± SD. *P<0.005; paired t-test. There is no significant difference between wt and sna, GFP, p35. (TIF) [file pgen.1007167.s002.tif]

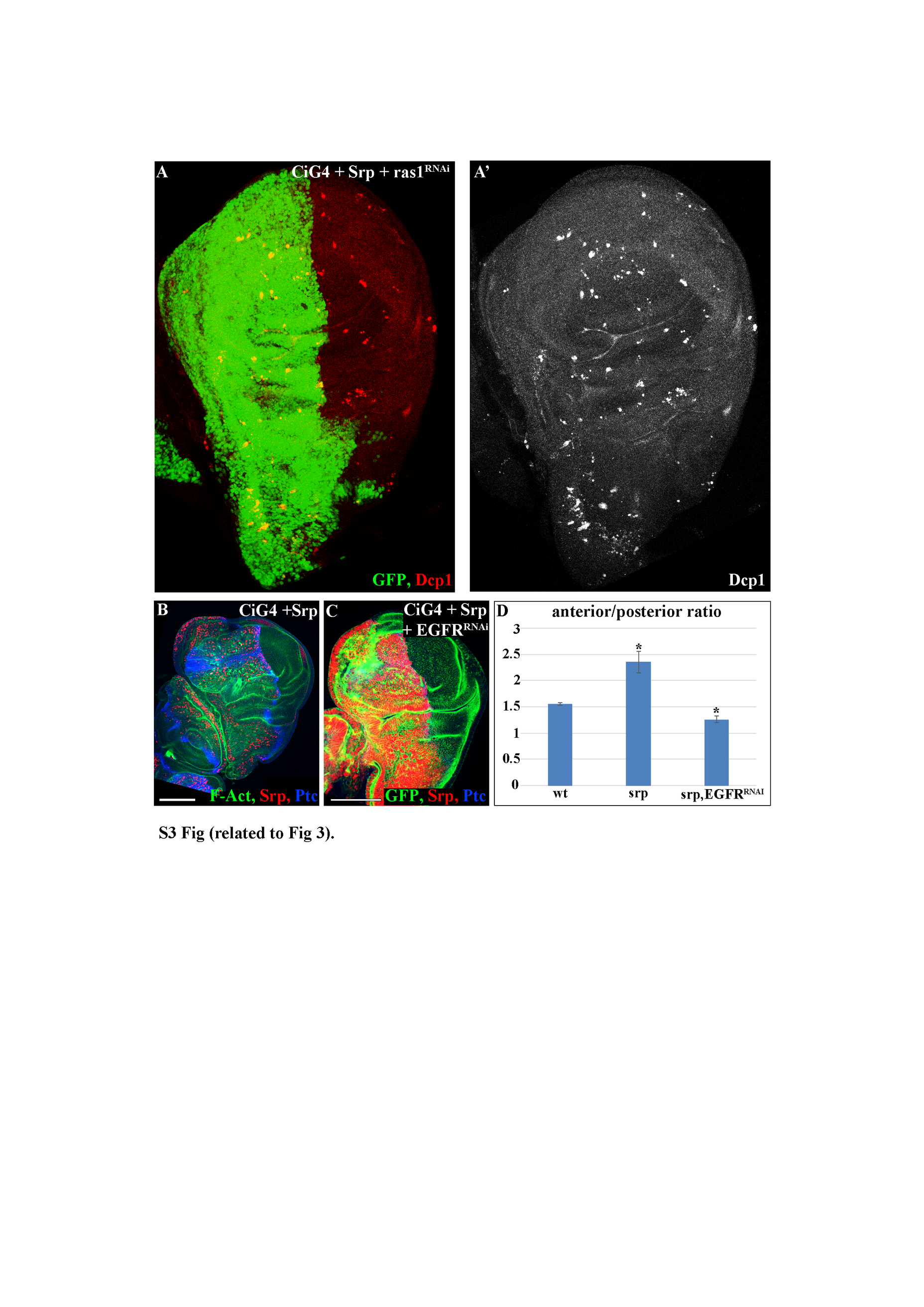

Supplement: S3 Fig — A-C 3rd instar wing discs from either individuals expressing GFP to mark anterior the anterior compartment together with Srp (A, C) and RasRNAi (A); or just Srp (B). Transgenes are under the control of the ci-Gal4 driver and tub-Gal80TS and the discs are fixed 48 hours after shifting to 29°C. A Staining for Dcp1 to visualise cell death. D. Histogram plotting the A/P width ratio of wing primordia expressing either GFP alone (wt), Srp alone (srp) or Srp together with EGFRRNAi. Data are presented as mean ± SD. *P<0.005; paired t-test. (TIF) [file pgen.1007167.s003.tif]

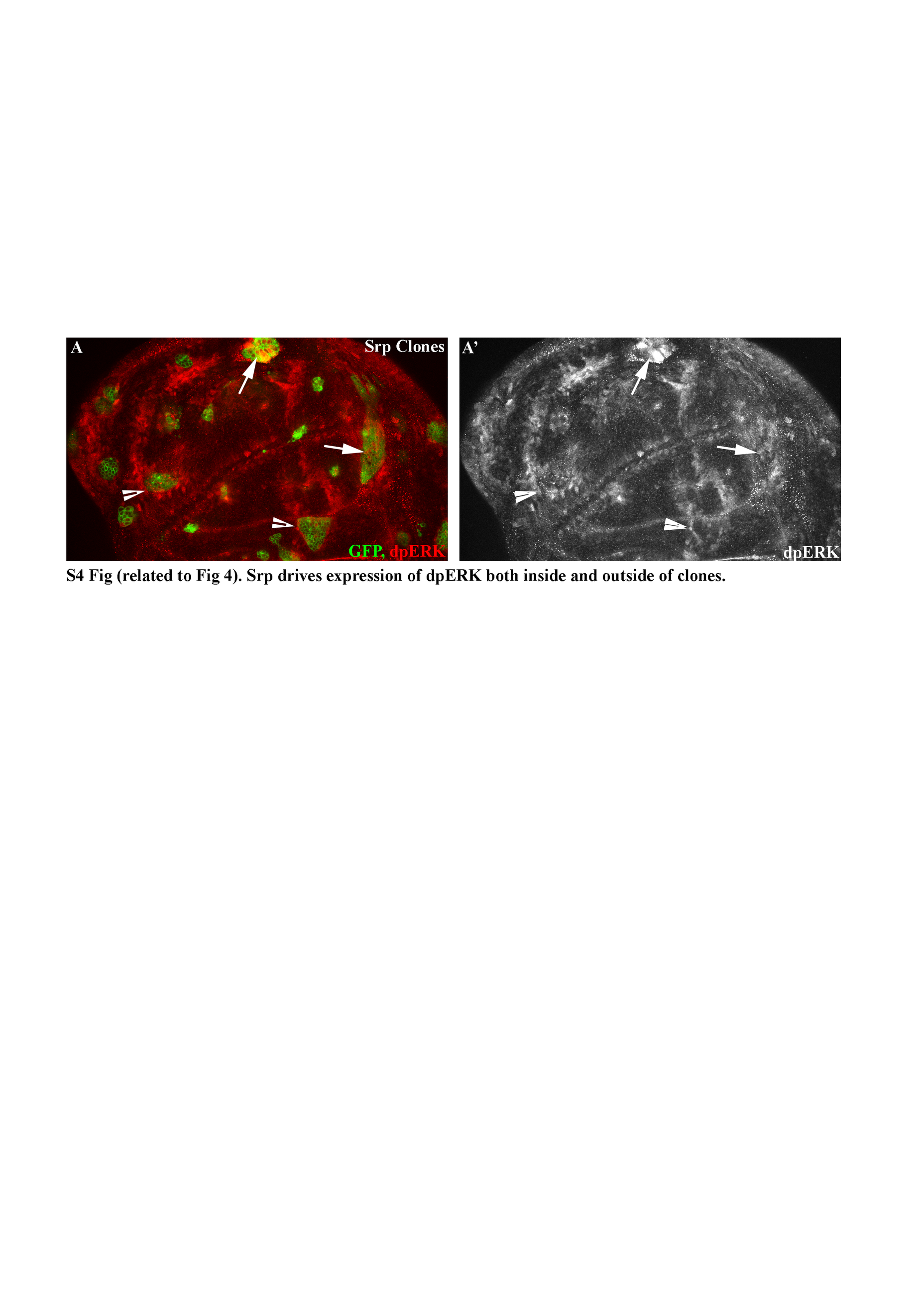

Supplement: S4 Fig — (A) Clones of UAS-srp were generated in an otherwise wild type background. Clones were induced by a 30 min heat shock and fixed after 24 hours. Staining for dpERK shows that Ras signalling is activated both inside (arrow) and outside (arrowhead) clones. (TIF) [file pgen.1007167.s004.tif]
